# Chloroform *versus* Ether

**Published:** 1891-09

**Authors:** W. H. Harsant

**Affiliations:** Surgeon to the Bristol Royal Infirmary


THE BRISTOL

Medico-Chirurgical Journal.

SEPTEMBER, 1891.

### CHLOROFORM *versus* ETHER.

BY
W. H. Harsant, F.R.C.S. Eng.,
*Surgeon to the Bristol Royal Infirmary.*

Scotland has always been prominent in everything relating to the study of anæsthetics; and the Glasgow Medico-Chirurgical Society has done good service in devoting three evenings to a discussion on this subject, and in publishing a full report of the proceedings in a good-sized pamphlet.[1]

A discussion on anæsthetics also took place at the recent meeting of the British Medical Association at Bournemouth; and these discussions serve a useful purpose in drawing attention to the present unsettled state of opinion on the subject. At both places the matter was treated from two points of view: that of experiment on animals and that of clinical observation, and from both these standpoints widely different views were expressed by different observers.

[1] *Discussion on Anæsthetics.* Edited by J. Walker Downie, M.B. Reprinted from the *Glasgow Medical Journal*, 1890–1.

The principal experimenters on the physiological action of anæsthetics within a recent period have been:—

1. The Glasgow Committee on Anæsthetics of 1879.
2. The Hyderabad Commission.
3. Dr. Macwilliam of Aberdeen.
4. Professor Wood in the University of Pennsylvania.

Two important questions, among others, have been considered by these authorities:

1. The comparative fatality of ether and chloroform.
2. The mode in which they kill—whether through the circulation or through the respiration.

Inasmuch as these two questions depend very much upon one another for their solution, they are generally considered together.

The two opposing views have long been looked upon in this country as those of Edinburgh and London respectively. Syme taught that we should be guided as to the effect of chloroform, not by the circulation, but entirely by the respiration; and this teaching was adopted by Lister, who did not consider it necessary to watch the pulse when giving chloroform, but recommended close attention to the respiration.

On the other hand, Erichsen, who may be looked upon as representing, in this respect, the teaching of the London schools generally, says that the finger of the administrator should never be off the pulse, but that breathing and circulation should both be watched.

Each of these views has now been backed up by the experiments of a great Commission. The Glasgow Committee of 1879 found, as the result of their experiments upon dogs and rabbits, that, apart altogether from the respiratory centres, chloroform has a disastrous effect on the heart. The Hyderabad Commission, on the other hand, as the result of a large number of experiments on the pariah dogs of India, and many others on monkeys and other animals, report that in every case where chloroform was pushed, the respiration stopped before the heart. They point out that chloroform, when given continuously, freely diluted with air, causes a *gradual* fall in the mean blood-pressure, provided the animal's respiration is not impeded in any way, and it continues to breathe quietly without struggling or involuntary holding of the breath. As this fall continues, the animal first becomes insensible, then the respiration gradually ceases, and, lastly, the heart stops beating. *But however concentrated the chloroform may be, it never causes sudden death from stoppage of the heart.* They further report that chloroform has no power of increasing the tendency to either shock or syncope during operation; that it in no way *per se* endangers a fatty heart; but, on the contrary, by lowering the blood-pressure, lessens the work that the heart has to perform, which is a positive advantage.

These two Commissions may be looked upon as the most important authorities on the question. But two other sets of experiments have been lately made which deserve attention. These are, firstly, those of Professor Macwilliam of Aberdeen, who made a series of experiments upon animals, chiefly cats, between 1888 and 1890, and came to the following conclusions among others :—

1. During chloroform anæsthesia the blood-pressure is lowered and the heart's action is weakened.
2. Dilatation of the heart commonly occurs also.
3. The tone of the heart-muscle is depressed, the cardiac walls become relaxed, and the functional efficiency of the heart is impaired.

4. Cardiac failure sometimes occurs in this way before the respiration stops.
5. That ether does not cause dilatation of the heart, nor a weakening of its action.

And, secondly, those of Professor Wood of Pennsylvania, who gives his opinion, as the result of numerous experiments on dogs in America, that chloroform is a cardiac paralysant, and often kills by a direct action upon the heart or its contained ganglia. He does not impugn the accuracy of the Hyderabad experiments, but suggests that difference in climate may account for the difference in result.

But in reply to these, Surgeon-Major Lawrie, who was the President of the Hyderabad Commission, tells us that the Glasgow Committee in 1879 and Professor Macwilliam in 1890 drew conclusions from an inadequate number of experiments. The Glasgow Committee made their observations on less than half-a-dozen animals; Professor Macwilliam made his on 75 cats; while the Hyderbad Commission did not discover the key to the safe administration of chloroform until they had made observations upon 565 animals and had reached their one hundred and forty-eighth manometer experiment. He challenges the Glasgow Committee, or Professor Wood, or Professor Macwilliam, to produce either an irregular trace or a dangerous dilatation of the heart with chloroform, before stoppage of the respiration, in any animal whose breathing is regular throughout the administration.

The point of difference then between these experimenters is, whether chloroform sometimes kills by its action upon the heart or circulation, without anything first going wrong with the respiration, or whether the patient is perfectly safe so long as the respiration goes on quietly and regularly. All are agreed that the *principal* danger lies with the respiration; but while the Hyderabad Commission would lead us to think that *all* danger lies in this direction, the other experimenters, however much they may differ in minor matters, are agreed that a certain degree of danger lies with the heart primarily. And upon this question hinges the other question—whether ether is a safer anæsthetic than chloroform; for all who insist upon the danger to the heart from chloroform are agreed that this danger is very much lessened by the use of ether, and they recommend ether accordingly as the safer anæsthetic; while the Hyderabad Commission, on the other hand, say that chloroform is quite as safe as ether: indeed, Dr. Lauder Brunton goes so far as to say it is safer, inasmuch as it is more certain in its action.

All this difference of opinion among physiologists is very unsatisfactory, and the practical anæsthetist is almost compelled to come to the conclusion that experiments upon animals do not give much assistance, and that he must rely on clinical observation.

But if we turn here, we find no less difference of opinion.

In Scotland, and especially in Edinburgh, the teaching of Syme still prevails, and a confident belief in the safety of chloroform is fully maintained. It has even been said that deaths from chloroform never take place in Edinburgh, and that it must be from some fault in the administration that deaths are so frequent elsewhere. In Germany, and throughout the Continent, scarcely any other anæsthetic than chloroform is employed; while in India, Surgeon-Major Lawrie tells us that this anæsthetic is now given as many times as throughout the whole of Europe. In America, on the other hand, ether has almost entirely replaced chloroform, and a general opinion prevails there that it is a much safer anæsthetic; while in England, as well as Ireland, there is a very growing opinion, especially among hospital surgeons and specialists in anæsthetics, that ether is safer than chloroform.

At the Bournemouth meeting, Dr. Dudley Buxton read a valuable paper, in which he pointed out that the conclusions of the Hyderabad Commission could not be applied to clinical work, and were calculated, therefore, to do harm. He was strongly of opinion that ether was a safer anæsthetic than chloroform, and he recommended close attention to the pulse and heart-beat as well as the respiration. A similar view was taken by Mr. George Eastes and other speakers who followed, and especially by Mr. Pridgin Teale, who in his well-known forcible manner explained the proper mode of administering ether, and pointed out that nearly all the supposed objections to its use might be obviated by attention to a few simple rules of administration. He expressed his preference for Clover's portable inhaler, as being the best we now possess; but suggested that the india-rubber air bag supplied with it was often made too small. He advocated procedure by "harmonic progression," taking the first half-minute to let the patient get accustomed to the machine, the next half-minute to let his sensibilities become numbed, and then to push the ether as rapidly as possible; by which means it would rarely take five or six minutes to put the patient under, and would often take less than two minutes.

The principal advocate for chloroform, at this discussion, was Dr. Lauder Brunton, who gave a short account of his Hyderabad experiments, and recommended that Syme's directions should be closely followed in giving chloroform; viz., to use only chloroform of the best quality, to give plenty of it, and to give plenty of air with it.

With reference to the quality of the chloroform, an interesting point was brought forward by Dr. Copeman, Research scholar of the British Medical Association, who had found, in experimenting upon monkeys, that methylated chloroform, *i.e.* chloroform prepared from methylated alcohol, frequently caused death when pure chloroform did not; the moral of which is, that we should never risk methylated chloroform in using it on human patients.

It might be thought that the best way to settle the question of the comparative safety of ether and chloroform would be by considering the death statistics of each; but it is, unfortunately, very difficult to obtain trustworthy figures on a large scale.

Lyman gives, as the result of a summing-up of all the cases he can find:

| | | | | | |
|---|---|---|---|---|---|
| Ether - - | 99,255 inhalations, | with 6 deaths, | or 1 | in | 16,542. |
| Chloroform - | 492,235 ,, | ,, 84 ,, | ,, 1 | ,, | 5,860. |

Surgeon-Major Lawrie says that he and Syme conjointly have administered chloroform 45,000 times without a death.

Roger Williams, in a letter in the *Lancet* of February 8th, 1891, shows that the London mortality for chloroform gives 1 death in every 1,236 administrations.

Dr. Coats gives 12,000 administrations of chloroform in the Western Infirmary of Glasgow since 1874 with 5 deaths, or 1 in 2,400.

In St. Bartholomew's Hospital, during the last fifteen years, chloroform was given 17,666 times with 12 deaths, or 1 in 1,472; and ether 7,493 times with 1 death.

At the Manchester Royal Infirmary there have been no less than 5 deaths under chloroform since last January.

During the last seventeen years an accurate record has been kept of the anæsthetics administered in the Bristol Royal Infirmary, and during that time chloroform, or what is practically the same thing, a mixture of chloroform and ether, has been given 5,902 times with 3 deaths, and ether 704 times with 1 death; giving a rate of 1 in 1,967 and 1 in 704 respectively.

The following is a brief report of the fatal cases, for assistance in preparing which I am indebted to Dr. James Swain:

CASE 1.—*Death during administration of Chloroform.*—A man, aged 54, was placed on the operation table on December 30th, 1881, for the relief of a strangulated inguinal hernia. His pulse was very weak but quite regular, tongue moist, aspect rather pinched. Chloroform was administered on a flannel mask; and after patient had inhaled three small quantities, probably less than a drachm and a half in all, he began to struggle, and vomited some fæcal-smelling fluid. Loud tracheal râles were heard, and the breathing became embarrassed. He was turned on to his side, when he ejected about two quarts of fluid from the stomach. The pupils dilated, the pulse failed, and he became cyanosed. Artificial respiration was performed, and the tongue drawn forward; the air entered the lungs, and the pupils became small again. Vomiting, however, recurred profusely; and although he was inverted, and air was heard to enter the lungs in artificial respiration, yet it was not successful. At the *post mortem* examination, some food was found in the larynx just below the rima glottidis; the lungs were œdematous and sodden; the kidneys were granular; but the heart appeared healthy.

CASE 2.—*Death during administration of Chloroform.*—A woman, aged 45, was placed under chloroform on April 20th, 1883, for the purpose of a vaginal examination. She had taken fluid food a short time previously, and was subject to a chronic cough. About twenty drops of chloroform were given on a flannel mask, and afterwards a mixture of one part chloroform and two parts ether was used. She took the anæsthetic well, became unconscious in three minutes, was kept under the influence for ten minutes further, when the respiration grew shallow, the face became pale, and after two or three feeble respirations the breathing and pulse both ceased. Inversion and artificial respiration were used without avail. At the *post mortem* examination, tubercular peritonitis was found. The right lung contained a small cavity in its upper lobe, with tubercular deposits around; the left lung also showed evidence of tubercular deposits in its upper part. The heart was soft and flabby, but the valves were healthy. The first part of aorta was atheromatous. Brain and kidneys healthy.

CASE 3.—*Sudden Death after administration of Ether.*—A man, aged 24, was placed on the operation table on October 29th, 1884, for the purpose of having an incision made in a node on the tibia. He took the ether well, about an ounce and a half being used. He became quite conscious before being removed from the table, and was then carried back to the ward and placed in bed. He talked quite freely to the other patients, and remained quite conscious for half-an-hour; and then turned over on his side, became blue in the face, gasped for breath, and died in two minutes. There was no vomiting throughout. At the *post mortem* examination, the vessels of the brain were very full of blood, and the sinuses distended; but no embolism or hemorrhage could be discovered. The heart was normal, rather pale, but contracted, with small *ante mortem* clot. The lungs were both acutely congested, and almost black in colour. Other organs healthy.

CASE 4.—*Death from a mixture of Chloroform and Ether.*—A man, aged 50, was being operated on for cataract on May 19th, 1887. A mixture of chloroform and ether was administered on a flannel mask; and the patient suddenly died, apparently from cardiac failure.

No *post mortem* examination allowed.

Dudley Buxton very truly points out that we may learn quite as much from "anxious cases" as from deaths. It fortunately falls to the lot of few of us to see an actual death from an anæsthetic; but we have all of us seen many cases where life has seemed to hang in the balance, and it is from these cases that we learn far more than we do from the *post mortem* table. If chloroform be the anæsthetic employed, how common it is to find a sudden fall in the force of the pulse, a pallor of the countenance, and everything indicating failure of the heart's action, even while respiration is proceeding quite evenly and regularly. If in such a case as this we adopt the method, first taught us by Nélaton, of inverting the body, we find the colour return to the cheeks and the force to the pulse; and what possible explanation can be given to these phenomena except that the chloroform was acting as a cardiac depressant?

On the other hand, when ether is the anæsthetic employed, such occurrences as these are unknown: the face always remains a good colour, and the force of the pulse vigorous; in fact, almost the only danger we have to guard against is that of asphyxia; and this gives us a feeling of security which we never feel with chloroform.

The effect of the Hyderabad Commission will doubtless be to give an impetus to the use of chloroform; but whether this will be for good or ill, time will prove. The fear is that it will give a sense of false security to the great body of medical men, who naturally prefer the simpler anæsthetic, and it will prevent them from feeling the necessity of using the one more difficult to administer, though less dangerous in its results.

